# Regulation of FGF-2, FGF-18 and Transcription Factor Activity by Perlecan in the Maturational Development of Transitional Rudiment and Growth Plate Cartilages and in the Maintenance of Permanent Cartilage Homeostasis

**DOI:** 10.3390/ijms23041934

**Published:** 2022-02-09

**Authors:** Anthony J. Hayes, John Whitelock, James Melrose

**Affiliations:** 1 Bioimaging Research Hub, Cardiff School of Biosciences, Cardiff University, Cardiff CF10 3AX, Wales, UK; hayesaj@cardiff.ac.uk; 2 Graduate School of Biomedical Engineering, Faculty of Engineering, University of New South Wales, Sydney, NSW 2052, Australia; j.whitelock@unsw.edu.au; 3 Raymond Purves Bone and Joint Research Laboratory, Kolling Institute of Medical Research, Northern Sydney Area Local Health District, St. Leonards, NSW 2065, Australia; 4 Sydney Medical School, Northern, Faculty of Medicine and Health, The University of Sydney, Royal North Shore Hospital, St. Leonards, NSW 2065, Australia

**Keywords:** perlecan, cartilage homeostasis, development, Wnt, Hh, chondrocyte proliferation, chondrocyte differentiation, *Snail-1*, FGF, skeletogenesis

## Abstract

The aim of this study was to highlight the roles of perlecan in the regulation of the development of the rudiment developmental cartilages and growth plate cartilages, and also to show how perlecan maintains permanent articular cartilage homeostasis. Cartilage rudiments are transient developmental templates containing chondroprogenitor cells that undergo proliferation, matrix deposition, and hypertrophic differentiation. Growth plate cartilage also undergoes similar changes leading to endochondral bone formation, whereas permanent cartilage is maintained as an articular structure and does not undergo maturational changes. Pericellular and extracellular perlecan-HS chains interact with growth factors, morphogens, structural matrix glycoproteins, proteases, and inhibitors to promote matrix stabilization and cellular proliferation, ECM remodelling, and tissue expansion. Perlecan has mechanotransductive roles in cartilage that modulate chondrocyte responses in weight-bearing environments. Nuclear perlecan may modulate chromatin structure and transcription factor access to DNA and gene regulation. Snail-1, a mesenchymal marker and transcription factor, signals through FGFR-3 to promote chondrogenesis and maintain Acan and type II collagen levels in articular cartilage, but prevents further tissue expansion. Pre-hypertrophic growth plate chondrocytes also express high Snail-1 levels, leading to cessation of Acan and CoI2A1 synthesis and appearance of type X collagen. Perlecan differentially regulates FGF-2 and FGF-18 to maintain articular cartilage homeostasis, rudiment and growth plate cartilage growth, and maturational changes including mineralization, contributing to skeletal growth.

## 1. Introduction

Perlecan is a modular, multifunctional, instructive, cell regulatory proteoglycan with roles in a diverse range of physiological processes [1,2,3,4]. Perlecan promotes extracellular matrix (ECM) organization, stabilization and cellular attachment [2] through interactions with a range of cell adhesive glycoproteins to promote cell proliferation, differentiation and tissue development mediated by interactions with integrins, growth factors and morphogens [3] (Table 1).

Many of these interactions are mediated by the glycosaminoglycans (GAGs) of perlecan domain-I [4]. The fibroblast growth factor (FGF) family is a major group of growth factors regulated by perlecan through FGFR-1 and FGFR-3 in chondrocytes [5,6]. Perlecan also interacts with type II, III, IV, V, VI, XI, XIII, XV, XVIII collagen [7,8,9,10] forming cell-ECM interconnections that facilitate mechanotransductive communication and stabilization of the pericellular matrix (PCM). The aim of the present study was to assess how perlecan differentially promoted chondrogenesis in rudiment cartilage and regulated cell proliferation, matrix deposition, chondrocyte maturation, and the expansion of growth plate cartilage during endochondral ossification and skeletogenesis. This contrasts with the permanent articular cartilages that do not undergo morphological transitional changes like those seen in other cartilages; matrix synthesis and cell proliferation are repressively controlled by *Snail-1* and *Wnt* cell signalling to maintain the permanent cartilages in a homeostatic state to maintain articulatory properties in diarthrodial joints.

### The HS-Proteoglycans of Cartilaginous Tissues

**Figure 1:**
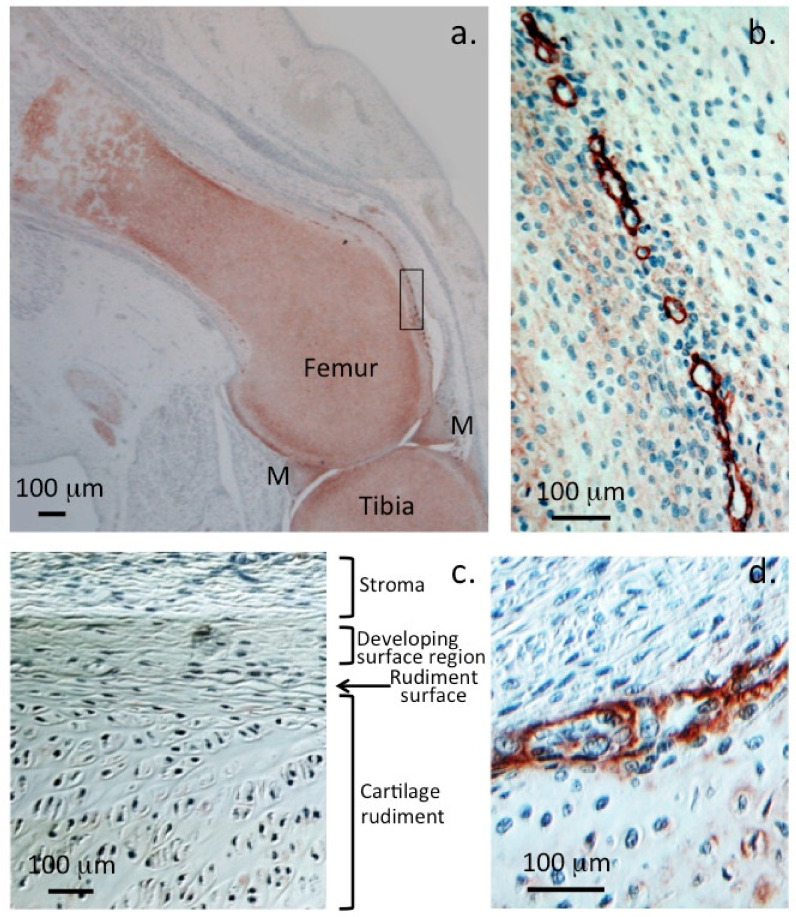
Localisation of perlecan in human foetal knee joints (12 weeks gestational age). Immunolocalisation of HSPG2 with perlecan domain-1 MAb A76 in a 12-week-old gestational age human foetal knee demonstrating perlecan as a major extracellular matrix proteoglycan of the tibial and femoral cartilaginous rudiments (**a**) and menisci (M). Perlecan is also prominently localised around the margins of small chondroprogenitor cell niches in the stromal tissue surrounding the rudiment (**b**). Nomarski differential interference contrast images demonstrate the differing stromal, surface, and central cartilaginous rudiment cell morphologies (**c**). Detail of a chondroprogenitor niche at the interface of the stromal and rudiment surface with perlecan prominently located around the niche (**d**). Chromogen NovaRED, nuclei stained with haematoxylin. Photosegments modified from [16] with permission © Melrose 2016.

Chondrocytes contain a number of ECM and cell surface and intracellular granular HS-PGs. Perlecan is the most abundant cartilage HS-PG; type XVIII collagen and agrin are also ECM cartilage HS-PGs [11]. Two families of transmembrane cell surface HS-PGs, the syndecans and glypicans, are also present on the chondrocyte cell surface. Serglycin has also been observed as a granular component within chondrocytes [12,13]. Cartilage thus has several potential sources of HS other than perlecan. Even so, development of the HS deficient perlecan exon 3 null mouse shows that perlecan HS has important roles to play in cartilage, tendon and IVD biology. Even in the presence of these other potential sources of HS [14,15], these HS-PGs thus do not compensate for HS depletion in the perlecan exon 3 null mouse. Perlecan is found in small chondroprogenitor stem cell niches in fetal cartilage rudiments [16] and these have roles in the development of diarthrodial joints, expansion of the fetal cartilages and establishment of primary and secondary ossification centres which are precursors to the development of growth plate cartilage [17] (Figure 1).

## 2. Perlecan Regulates Cartilage Transformation during Skeletogenesis

**Figure 2:**
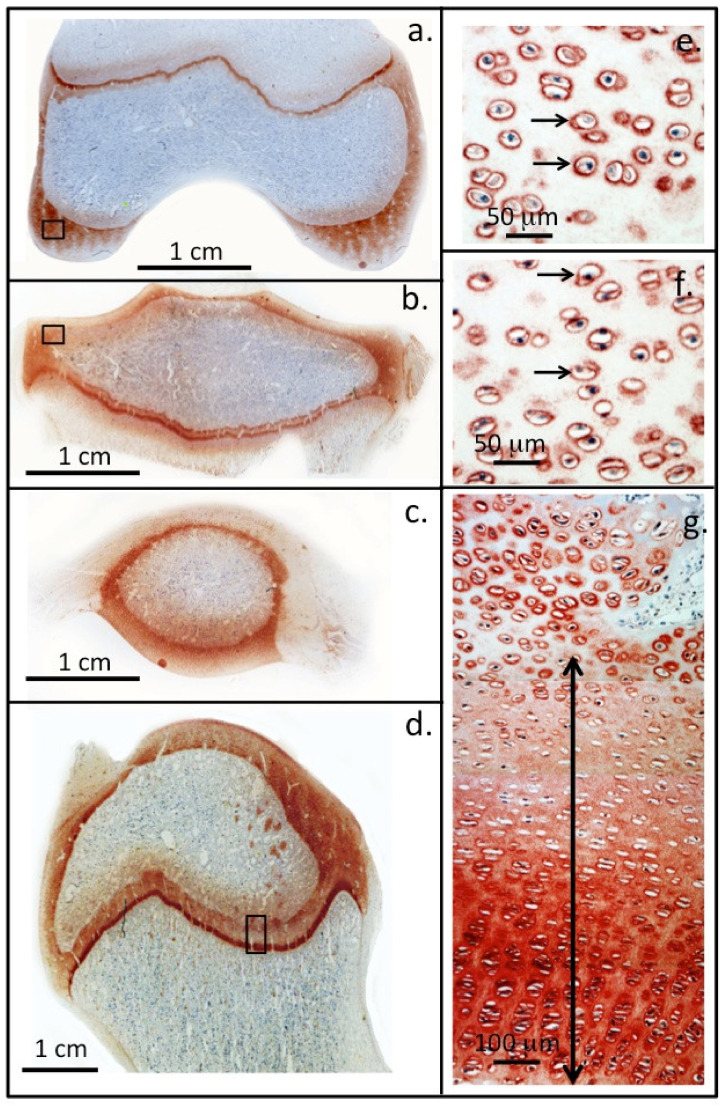
Perlecan localisation in ovine knee and hip joints. Immunolocalisation of perlecan in cartilaginous tissues of a two-year-old ovine knee femoral condyle (**a**) and tibial plateau (**b**), patella (**c**) and in the humeral head of a hip joint (**d**). Higher power images demonstrate the pericellular localisation of perlecan (small arrows) around chondrocytes in regions of the femoral (**e**) and tibial articular cartilages (**f**) (boxed areas in (**a**,**b**)). Perlecan is also present as a gradient throughout the femoral long bone growth plate ECM of the hip in the resting and proliferative zones (double headed arrow) and is prominently expressed pericellularly by the hypertrophic columnar hip chondrocytes located in the bottom of photosegment (**g**). NovaRED chromogen, perlecan localised with MAb A7L6 to perlecan domain IV. Photo segments (**a**–**g**) modified from [9] reproduced under Open Access Creative Commons Attribution 4.0 International licence images © the authors (2010).

Perlecan is a critical ECM proteoglycan in cartilage development and has roles in cartilage biomechanics in mature cartilage [17,18,19,20]. A scheme is provided to explain the stages of cartilage development we reviewed. Perlecan has a pericellular localisation pattern in the permanent articular cartilages but forms extracellular gradients in the growth plate cartilages (Figure 2). Perlecan deficiency leads to defective pericellular matrix formation [21,22], reduced cellularity and cartilage stiffness in vivo [21], predisposing this tissue to the early onset of osteoarthritis (OA), joint deformities, and chondrodysplasia [22,23,24,25]. Perlecan is a chondrogenic proteoglycan displaying an almost identical distribution in developmental cartilaginous tissues as type II collagen and aggrecan, and actively supports sequential chondroblastic differentiation, chondrogenesis, matrix deposition, and expansion and maturation of the developmental cartilage rudiment templates [8,17]. In the permanent articular cartilage, cell proliferation and matrix expansion are held in check to achieve tissue homeostasis [26]. However, this is not the case in the growth-plate cartilages that undergo morphological change during tissue maturation with ECM composition varying as the cartilage transitions to bone during endochondral ossification or the cartilage rudiments that are transient developmental scaffolds in skeletogenesis [8,26]. HS-PG synthesis is deregulated in human OA cartilage [27], affecting FGF signalling and the maintenance of cartilage composition and functional properties. FGF-FGFR signalling is dictated by specific HS saccharide sequences on the side chains of perlecan [28,29].

**Scheme to explain the component parts of this review**. (Each of these component parts are discussed in detail later in the review).

**Foetal rudiment cartilage:** a developmental template for diarthrodial joint development and the formation of cartilage masses that will eventually form the bony skeletal elements. Stem cell niches in the surface regions of the rudiments surrounded by perlecan establish pluripotent migratory chondroprogenitor stem cell lineages that co-ordinate joint cavitation and growth of the cartilage rudiment.

Perlecan is an extracellular proteoglycan in the rudiment and directs chondrocyte proliferation and the laying of cartilaginous tissue in the rudiment cartilages promoting co-ordinated tissue expansion. FGF-2 and FGF-18 signal through perlecan to promote chondrogenesis in the rudiment.

Differentiation of rudiment chondrocytes leads to hypertrophy and establishment of the primary and secondary ossification centres that will eventually become the growth plate cartilages. Perlecan and FGF-18 are highly expressed by the hypertrophic cells surrounding the ossification centre. FGF-18 promotes a shift from chondrogenesis to osteogenesis by cells in the centre of the ossification centres and this region undergoes mineralization.

**The permanent cartilages**. Surface regions of the cavitated rudiment cartilages are spared developmental changes and these will become the weight bearing articular cartilage of the mature joint. Perlecan maintains the homeostasis of these cartilages by controlling FGF-2 and FGF-18 growth factor bioavailability minimising cell division, excessive matrix deposition and tissue expansion. The permanent cartilages turn-over slowly but are maintained in an optimal weight bearing form to participate in joint articulation. Perlecan is a prominent pericellular component surrounding articular chondrocytes and maintains articular chondrocytes viability and tissue homeostasis.

**The growth plate cartilage**. The cartilage rudiments containing primary and secondary ossification centres undergo further maturational changes to form the growth plate cartilages. Perlecan is an extracellular proteoglycan and forms gradients in these tissues which with sequestered FGF-2 and FGF-18 promotes chondrocyte proliferation and differentiation into characteristic columnar arrangements of growth plate chondrocytes. With further proliferation the growth plate chondrocytes reach maturity and undergo hypertrophy, perlecan and FGF-18 are upregulated in these cells but aggrecan and type II collagen production ceases and type X collagen appears and this cartilage mineralizes as part of the endochondral ossification process.

### 2.1. Changes in Perlecan-HS Fine Structure Associated with Cartilage Degeneration

OA, characterized by cartilage loss and subchondral bone remodelling, occurs due to abnormal mechanical loading which generates characteristic lesions in the weight-bearing regions of articular cartilage not protected by the menisci in diarthrodial joints [30]. HS-PGs bind to many cartilage proteins that stabilize cartilage and maintains its functional properties and tissue homeostasis. Over 400 bioactive HS binding proteins have been identified [31,32,33], which include growth factors, morphogens, structural and cell attachment glycoproteins, proteases, and protease inhibitors. Assessment of the GO (gene ontology) biological process categories and KEGG (Kyoto Encyclopedia of Genes and Genomes) pathways enriched in heparin/HS interactome members demonstrates the biodiverse interactive properties and biological processes that HS-PGs regulate [33,34]. HS-PGs modulate the localization, retention, and biological activity of many components in cartilage [27]. Surprisingly, a decreased HS content or reduced HS sulphation levels in cartilage is chondroprotective, protecting against OA progression by regulating protease activity. In a study by Severmann and colleagues, reduced HS levels, reduced HS sulphation and alterations in HS fine structure were all observed in OA cartilage which had decreased degenerative OA-histopathology scores [35]. Increased levels of 6-*O*-sulphation in OA cartilage, due to increased expression of HS6ST1, a 6-*O*-sulphotransferase, and GLCE (glucuronyl C5-epimerase), which promotes epimerization and inversion of *D*-GlcA structure to *L*-IdoA and 2-*O*-sulphation, may contribute to the OA process. 6-*O* sulphation is a critical modification in HS and controls interactive properties with many ligands. *N*-sulphation of GalNAc by *N*-deacetylase/*N*-sulphotransferase (Ndst-1) follows the HS6TI and GLCE biosynthetic steps in HS assembly. An alteration in the fine structure of perlecan HS chains in Ndst1+/− mutant mice leads to a reduction in the degradation of aggrecan and collagens by matrix metalloproteases (MMPs). Inactivation of Ndst1, a gene that regulates high sulphation, results in the production of HS that is severely undersulphated. Homozygous Ndst1−/− mutants die during the early postnatal development due to respiratory failure; heterozygous Ndst1+/− animals, however, are viable and do not display phenotypic changes in HS structure. NDST removes acetyl groups from GlcNAc, replacing these with essential *N*-sulphate groups required for further HS modifications during subsequent biosynthetic steps; without *N*-sulphation, no *O*-sulphation or GluA to IdoA epimerization occurs. Ndst1−/− mice exhibit delayed endochondral ossification, shortened calcific limb zones, and delayed chondrocyte and osteogenic differentiation and proliferation due to decreased BMP-2 and -4 activity.

Elevated expression of perlecan occurs in OA [36] by small clones of adult stem cells which mount an attempted repair response to altered joint mechanics. The PCM and its components (perlecan, collagen and hyaluronan) maintain the normal chondrocyte PCM microenvironment. Changes in the PCM produces signals that are detected by cell membrane receptors including Ca2+ channels, primary cilium, and integrins. These signal to downstream mechanotransductive molecular pathways that convert ECM signals to chemical and biological signals and mechanosensitive gene transcription [18,20,37]. FGF-18 is expressed by normal chondrocytes in articular, rudiment, and growth plate cartilage and is highly expressed by hypertrophic chondrocytes (Figure 2a) in the chondrocyte columns (Figure 2b,c). FGF-18 induces early chondrogenic differentiation of bone marrow stromal progenitor cells in pellet culture, producing a tissue of similar composition to mature human articular cartilage [38]. Downregulation in *Acan* and *Col2A1* expression then occurs on day 31 and a progressive shift to an osteogenic phenotype accompanied by Ca2+ deposition in the pellet occurs and co-expression of the CS sulphation developmental motifs 4-C-3 and 7-D-4 and type X collagen (Figure 2d,e) [38]. In the same study, bone marrow stromal progenitor cells stimulated with FGF-2 did not progress to an osteogenic phenotype but a chondrogenic phenotype was maintained throughout the full duration of the study (up to 41 days culture). Thus FGF-2, FGF-18 and perlecan differentially regulate cartilage and bone development by chondroprogenitor cells.

**Figure 3:**
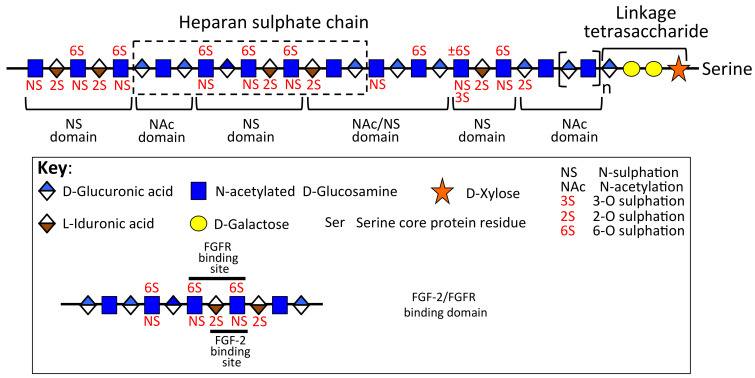
Schematic depiction of the domain organization of a hypothetical HS chain showing the FGF-2 and FGFR binding domain. Figure modified from [46,47].

HS sequence and chain length both regulate FGF-2 activity. FGF-2 interacts strongly with an epitope containing an IdoA2S-GlcNS-IdoA2S trisaccharide sequence (Figure 3); flanking 6-*O*-sulphation is also considered to be important in conferring specificity [38,39,40,41]. Perlecan forms a complex with FGF-2 and tyrosine kinase-containing FGFRs. The cell signalling induced by this interaction results in phosphorylation of extracellular signal-related kinase (ERK) and focal adhesion kinase (FAK), which promote cellular proliferation [42,43]. Besides binding to HS and forming complexes with FGFR-3, FGF-18 can also bind to perlecan domain III in the absence of GAG chains. FGF-18 co-ordinates chondrogenesis and osteogenesis [44,45] whereas FGF-2 promotes chondrogenesis only. FGF-18 knockout mice have defects in endochondral ossification, showing that FGF-18 is essential for long bone growth. FGF-18 thus has dual areas of action in chondrogenesis and osteogenesis of relevance to cartilage and bone development.

**Figure 4:**
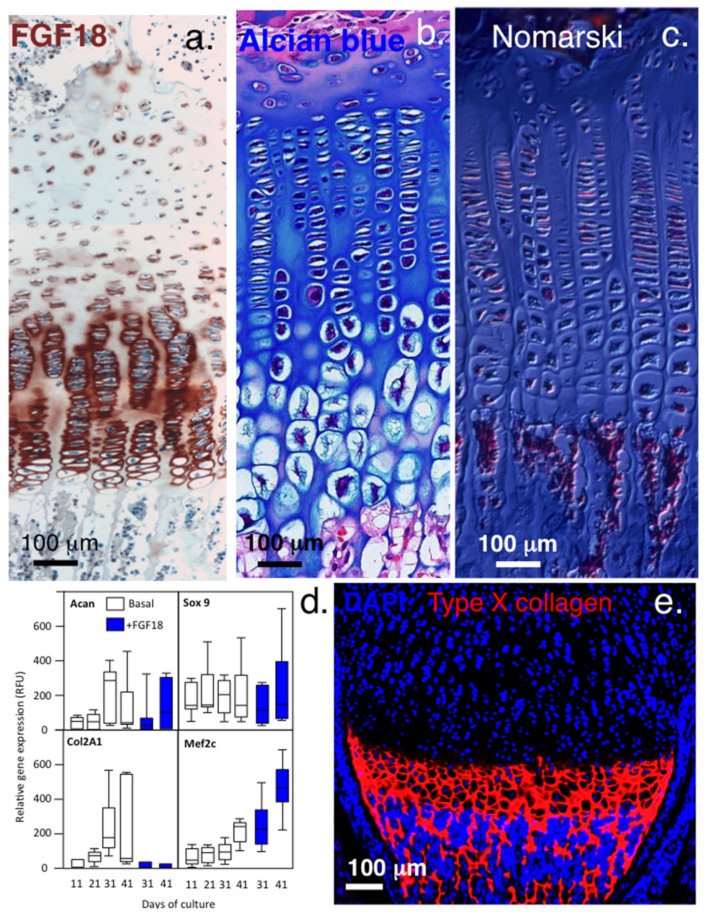
Immunolocalisation of FGF-18 and type X collagen in growth plate cartilage. Upregulation of FGF-18 immunolocalised in hypertrophic growth plate chondrocytes (**a**), with their enlarged morphologies clearly visualized by alcian blue staining (**b**) and in a Masson’s trichrome image viewed under Nomarski differential interference contrast optics (**c**). Gene profiling shows that FGF-18 initially stimulates chondrogenesis in bone marrow chondroprogenitor cells up to day 30 in micromass pellet culture but by day 31, type II collagen expression ceases and osteogenic differentiation (Mef2c) is initiated (**d**). FGF-2, however, maintains chondrogenesis throughout the full period of pellet culture (up to day 41). Type X collagen synthesis occurs in FGF-18-stimulated cultures from day 31 and is clearly evident at the chondro-osseous junction (**e**). FGF-18 also upregulates *Snail1* expression in the hypertrophic growth plate chondrocytes. Images (**a**–**d**) ©Melrose 2016, reproduced from [50] with permission. Images Image (**e**) supplied courtesy of DrYao Hao, © Yao Hao 2019, Institute of Genetic Medicine, International Centre for Life, Newcastle University, UK.

*Snail-1* promotes FGF signalling in pre-hypertrophic and hypertrophic growth plate chondrocytes, and uncouples the normal regulation of the permanent articular chondrocytes, repressing the transcription of *Aggrecan* and *Collagen 2a1* (Col2a1). Snail-1 is a major target of *Shh* signalling, promoting chondrocyte hypertrophy and ossification during chondrogenesis. FGF-18 and *Snail1* are expressed in hypertrophic chondrocytes during normal development, and by pre-hypertrophic chondrocytes. In the absence of *Snail-1*, signalling through FGFR-3 and chondrogenesis is abolished [48]. *Snail-1* acts downstream of FGFR-3 in pre-hypertrophic chondrocytes, where it inhibits chondrocyte proliferation and represses *Col2a1* and *Acan* expression through interactions with histone deacetylases 1 and 2 [48,49]. This promotes a downregulation in type II collagen expression (Figure 4d) and a switch to type X collagen synthesis promoting growth plate maturation (Figure 4e).

*Snail*-1 is a transcriptional effector of FGFR-3 signalling and downregulates type II collagen and aggrecan gene expression during chondrogenesis [48] and controls TGF-β responsiveness and the differentiation of mesenchymal stem cells (MSCs). The control of *Snail-1* expression is complex, occurring at multiple levels [51]. Retention of *Snail-1* in the nucleus is promoted by the protein kinases Lats2 (large tumour suppressor kinase 2), PAK1 (Serine/threonine-protein kinase) and ERK2 (Extracellular Signal-Regulated Kinase 2). *Snail-1* is expressed at different stages of embryonic development, however its expression is very restricted in adults once the growth plates undergo closure. In pellet cultures of bone marrow chondroprogenitor cells stimulated with FGF-18, downregulation of Acan and Col2A1 synthesis was seen after 31 days of culture, recapitulating changes seen in the hypertrophic chondrocytes of human growth plate with maturational development (Figure 4a,d). FGF-2 did not induce such changes and mimicked the permanent cartilages maintaining chondrocytes in a cartilage phenotype [50]. The repressive properties of *Snail-1* could potentially provide a mechanism whereby mature chondrocytes in the permanent cartilages downregulate type II collagen and aggrecan synthesis to maintain tissue homeostasis while the rudiment and growth plate chondrocytes undergo proliferation, active matrix production, and tissue expansion until they reach hypertrophy, where *Snail-1* activity then downregulates type II collagen and upregulates type X collagen production during terminal differentiation.

### 2.2. Roles for Wnt and Hh in Cartilage Development

Perlecan transports Wnt and Hedgehog (Hh) protein via the the LDL receptor of perlecan domain II [52,53,54]. Wnt and Hh are lipid modified proteins of limited solubility; formation of perlecan gradients is important for Wnt and Hh morphogen transport and provides regulatory input in the development of the rudiment and growth plate cartilages (Figure 2g). Perlecan has a strict pericellular localisation in articular cartilage (Figure 2e,f), and does not form ECM gradients in this tissue, thus the permanent cartilages do not undergo transitional morphogenesis like that found in the rudiment cartilages and the growth plates. While several Wnt family proteins are reported to promote degeneration of mature articular cartilage in osteoarthritis (OA) [55,56,57], 6-*O* sulphation of perlecan HS chains prevents Wnt protein interactions with Frizzled, its cognate receptor, preventing degenerative Wnt cell signalling. Desulphation of perlecan HS by Sulf-1 promotes Wnt signalling and degradative changes in mature articular cartilage, confirming the functional status of the perlecan–HS chains [58]. Wnt also regulates chondroprogenitor proliferentiation and differentiation in rudiment and growth plate cartilage development [53] and undergoes an interplay with BMPs in the regulation of rudiment, permanent, and growth plate cartilage [52]. Hh proteins regulate cartilage development [6] and a number of developmental processes along with Wnt are thus potential molecular targets in OA therapeutics [53,57,59]. In humans, nineteen Wnt protein morphogens establish concentration gradients that drive cellular migration, promoting tissue development [60]. Perlecan transports the Wnt in tissues, since these are poorly soluble, to establish these concentration gradients [61]. Wnt proteins are palmitoylated signalling glycoproteins that are poorly soluble in aqueous media, however they bind with high affinity to perlecan domain II which is a low-density lipoprotein receptor, thus perlecan is a Wnt transporter proteoglycan. Other transport proteins have also been identified which maintain the solubility of Wnt proteins and promote long-range signalling [62]. Wnt proteins can also act as growth factors for stem cells. β-catenin and perlecan regulate *Wnt* signalling in *Drosophila* and humans [3,63].

The Wnt pathway regulates mammalian cell proliferation, differentiation, and development. Mono-unsaturated fatty acyl chains in Wnt are critical for cell signalling, transport, and receptor activation [64]. The knee joint infrapatellar fat pad is not just a benign tissue, but participates in pathological processes [65,66]. In OA, the infrapatellar fat pad n3/n6 fatty acid ratio is disturbed, possibly effecting Wnt signalling processes in OA [67]. Target cells contain surface Frizzled and Smoothened receptors that are involved in the reception of Wnt and Hh signals at the cell surface. While Wnts and Hhs are unrelated proteins, they are both modified by lipids and their cell signalling pathways share similarities [68,69]. The *Hh* and *Wnt* signalling pathways are essential regulators of cell proliferation, differentiation, oncogenesis, embryogenesis, and cellular differentiation. *Hh* signalling acts upstream of the *Wnt* signalling pathway, and negatively regulates *Wnt* activity via secreted frizzled-related protein 1 (SFRP1). The Wnt/β-catenin pathway downregulates Hh activity, an imbalance in Hh and Wnt regulation can promote cancer development. The activation of SFRP1 is an important regulatory component of the two signalling pathways. As lipid modified proteins, Wnt and Hh both have limited solubility, thus their interaction with perlecan is important for their transport in tissues and the establishment of concentration gradients which affect tissue development and morphogenesis [61]. Remodelling of HS-PGs in the tumour microenvironment can thus regulate tumour-promoting interactions involving Wnt/Hh [70], demonstrating a further aspect of perlecan’s instructive properties that can be modified by proteolytic processing in situ [1]. Disruption of this process has been proposed as a potential therapeutic anti-tumour targeting strategy [64].

## 3. Perlecan’s Differing Properties in Permanent, Rudiment, and Growth Plate Cartilage

As already discussed, the reason why tissues displaying an ECM distribution of perlecan undergo tissue expansion, cellular proliferation, and differentiation is due to the gradients of perlecan and growth factors/morphogens laid down in these tissues that drive tissue developmental processes [71,72,73,74,75,76,77]. This contrasts with tissues where perlecan has a strict pericellular distribution. In this case, growth factors are sequestered by the pericellular perlecan which controls their bio-availabilities sufficient to maintain the tissue in a homeostatic state, but insufficient to support cellular proliferation, differentiation, and the laying down of new ECM resulting in tissue expansion [26]. HS is an important functional determinant of perlecan responsible for the binding of a number of growth factors. Growth plate perlecan also contains chondroitin-4,6-disulphated residues in foetal tissues which regulate collagen fibrillogenesis by chondrocytes [78]. The GlcNAc-GlcA disaccharide is a repeat disaccharide that occurs in HS. The GlcNAc residues of HS are *N*-acetylated and *N*-sulphated at C2 and further sulphated at C6. GlcA also undergoes inversion in structure when it is converted to the IdoA epimer and sulphated at C2. C3 sulphated epitopes in HS exist, but are relatively rare. Unmodified *N*-acetylated disaccharide units (denoted NA domains) and highly modified sulphated regions (NS domains) also occur in the HS chain. HS-mediated FGF-2 signalling is determined by the structure of its highly sulphated domains distributed within the HS chain. The sulphation pattern on HS in charged microdomains acts as ligand docking sites. Specific binding structures for FGF-2, PDGF, lipoprotein lipase, and antithrombin have been determined [79]. An examination of the distribution of the NA and NS domains and 2-*O* and 6-*O* sulphation in human umbilical artery endothelial cell (HUAEC) perlecan, and in perlecan samples produced by a continuous endothelial cell line (C11STH) and colon carcinoma cell line (WiDr), showed similar 2-*O* and 6-*O* sulphation levels. The ability of these perlecan samples to bind FGF-2 and to induce proliferation of BaF32 B cells engineered to express specific FGFR isoforms differed, and the FGFR isoform is utilized in cell signalling [80]. Cell signalling through FGFR-1 initiates a catabolic response in cartilage [81]. However, FGFR-3 signalling is anabolic (Figure 1). Perlecan complexed with FGF2 is a chondrocyte mechanotransduction mitogenic signal mediated through activation of MAP kinases [82]. FGF-2 mediates diverse cellular processes including apoptosis, cell survival, chemotaxis, cell adhesion, migration, differentiation, and proliferation [83].

### 3.1. The HS Chains of Perlecan Can Promote or Inhibit Cellular Proliferation and Matrix Production

Cells dynamically regulate the structure of their pericellular HS-PGs in response to subtle ECM cues received by the cells during tissue development. Studies on tumour cells have shown that growth-promoting as well as growth-inhibiting cryptic HS sequences are contained within pericellular HS-PGs on the tumour cell surface. HS can promote or inhibit growth depending on how closely the HS structure mimics biologically active growth-promoting HS sequences. Cell surface HS can also engage HS receptors without activation of the receptor and cell signalling occurring, thus cell division is blocked and proliferation or cell migration does not occur [84]. Pericellular perlecan around chondrocytes in the permanent cartilages may contain cryptic HS inhibitory glycoforms that maintain a quiescent non-proliferative state that maintains tissue homeostasis. Such a proposal is not without precedent; perlecan also inhibits smooth muscle cell proliferation but in transgenic HS deficient mice, intimal hyperplasia and smooth muscle cell proliferation are increased. This inhibitory effect of smooth muscle cell perlecan resides in its HS side chains [85,86].

Removal of HS from cultured articular chondrocytes using heparanase promotes cell proliferation and matrix production [87], suggesting that chondrocytes in mature permanent cartilages do not actively proliferate or lay down matrix components or undergo cartilage expansion since they are surrounded by inhibitory HS sequences in their pericellular perlecan. IVD cells from *hspg2* exon 3 null mice which are deficient in HS display accelerated growth, reach hypertrophy earlier and elaborate ECM PGs to a greater extent compared to WT mice with normal HS levels [15].

HS biosynthesis and the enzymes responsible for the assembly of specific HS structures were determined a decade ago. Our understanding of how spatiotemporal tissue or cell-specific HS glycoforms are regulated in tissue development remains elusive. GAGs are multifunctional entities; it is important to develop molecular strategies to understand how various GAG glycoforms arise in tissues and how they are dynamically altered and regulated in tissue development [88]. It is not known if the HS chains of perlecan in the permanent articular cartilages and transitional rudiment and growth plate cartilages differ in some manner that could account for differences in cell proliferation, matrix deposition, and tissue expansion promoted by perlecan in these tissues. A study conducted in 2002 showed that three different cell sources (HUAEC, human arterial endothelial cells, a C11 STH endothelial cell line, and Widr colon carcinoma cells) produced perlecans with differing HS fine structure which affected their abilities to bind FGF-2 and activate FGFRs to mediate cell signalling and cellular proliferation. All of these perlecans bound FGF-2 but Widr perlecan did not activate FGFR1c-3c and did not induce cell proliferation. C11 STH perlecan signalled through FGFR1c but not FGFR3c. Only HUAEC perlecan signalled through FGFR1c and FGFR3c and elicited a cell proliferative response through these receptors. Thus, the HS fine structure can account for some of the observed differences in the functional properties of perlecan in different cartilage types.

#### 3.1.1. Variation in Perlecan Structure and FGF-18 Interactions Mediates Changes in Growth Plate Cartilage

The CS/HS composition and sulphation patterns in perlecan show spatio-temporal variation in the growth plate as it undergoes maturation leading to endochondral ossification, and has differing directive effects on the regulation of growth plate chondrocytes [89]. This is an aspect of perlecan HS chains which allows them to direct transitional tissue development. Perlecan isolated from the hypertrophic and lower proliferative zones of growth plate cartilage has been shown to contain larger CS chains and a different CS and HS disaccharide composition than perlecan isolated from the resting zone. Thus, perlecan turnover during maturation of the growth plate results in the replacement of resident perlecan by a form with a different sulphation pattern. Furthermore, FGF-18 differs from other FGFs in that it can induce cell proliferation in FGFRIIIc Baf32 cells with perlecan forms devoid of HS in domain I. To do this, FGF-18, like FGF-7, utilises a low-affinity binding site in perlecan domain III to promote cell proliferation [89,90]. Enzymatic removal of the HS and CS chains from growth plate perlecan has demonstrated FGF-18 binding sites in perlecan domain-III [89]. Thus, alterations in structure and sulphation of perlecan are important functional determinants in tissue morphogenesis.

#### 3.1.2. FGF2-Mediated Chondrocyte Signalling in Cartilage

FGF-2 is an important perlecan ligand in cartilage and acts as a mechanotransducer to the chondrocyte when cartilage is damaged or excessively loaded [91]. FGF-2 is chondroprotective, inhibiting the induction of aggrecanase in articular chondrocytes [92,93], and delays cartilage degradation in OA [14]. Mechanical signals to chondrocytes drive beneficial responses to maintain tissue homeostasis; modulation of specific cell signalling pathways offers a choice of strategies which might be employed to treat OA. Perlecan is a central cell signalling hub in these events and actively participates in mechanotransductive pathways in the pericellular matrix [24,91]. Atomic force microscopy (AFM) studies show perlecan has cytoprotective modulatory effects on the forces transmitted to the chondrocyte via the chondron type VI collagen network in tissues subjected to compressive and shear loading [19,94,95]. FGF-2 is an abundant pericellular component of articular chondrocytes. Inhibition of FGFR-1 using siRNA show FGFR-1 has roles mediating degenerative change in human adult articular chondrocytes. FGFR-1 activation by FGF-2 promotes catabolic changes in cartilage and impedes anabolic responses [81]. FGFR signalling occurs through the Ras/Raf/ERK and PI3 kinase/PDK/Akt cell signalling pathways.

#### 3.1.3. Differential Chondrocyte Signalling through FGFR1 and FGFR3

The form of perlecan synthesized by growth plate chondrocytes contains HS and CS side chains. HS interactions promote FGF/FGFR cell signalling, while the CS chains negatively regulate these interactions and may impede the release of FGFs from this complex [3]. VEGF isoforms also exhibit differential binding to the HS side chains of perlecan. This has a modulatory effect on VEGF signalling and creates a VEGF gradient at the cell surface that is important in tissue development [3]. The GAG types and sequence of the HS chains on perlecan are determinants of perlecan’s biological activity in situ. HS chains of perlecan synthesized by endothelial cells have been shown to bind to FGFR-3 IIIc (but not FGFR-1 IIIc), eliciting proliferative responses in FGFR-3c-expressing Baf32 cells [80]. Chondrocytes undergo differential cell signalling through FGFR1 and FGFR-3. FGFR-1 promotes catabolic effects in OA cartilage whereas FGFR3 promotes cellular proliferation and matrix production during tissue growth [96]. Inhibition of FGFR-1 expression in knee cartilage has been shown to inhibit cartilage degeneration [97]. FGFR-3 coordinates cartilage and bone development [98]. The response of FGF2 in cartilage is controversial since FGF-2 perlecan interactions can both promote catabolic events as well as anabolic tissue changes depending on which FGFR isoform is activated. In the permanent articular cartilage, activation of FGFR-1 and FGFR-3 are balanced and there is no net tissue loss or gain under homeostatic conditions when FGF signalling is efficiently regulated [96]. Tissue expansion and cell proliferation occurs when perlecan HS promotes cell signalling through FGFR-3, while cartilage degeneration occurs when signalling through FGFR-1 predominates.

## 4. Electrophysiological Events and Joint Mechanics Regulate Chondrocyte Metabolism

The chondrocyte is responsive to its ionic and biomechanical micro-environment [99,100]. In the last decade, several cell surface ion selective channels, pumps, or exchangers have been identified in chondrocytes with important cell regulatory properties [101,102,103,104,105]. Low metabolic activity in fully differentiated or senescent chondrocytes may contribute to the slow turnover of ECM components occurring in the permanent articular cartilages that maintains tissue homeostasis but inhibits cell proliferation, ECM remodelling, or tissue expansion, and may explain the low self-repair capability of adult articular cartilage. Chondrocytes are more metabolically active in the rudiment cartilages, which is self-evident from the rapid ECM deposition and cell proliferative events that lead to tissue expansion and maturational changes in tissue composition of the rudiment template, leading to the establishment of ossification centres that eventually lead to development of the long bones and growth plate cartilages through the endochondral ossification process [17].

Chondrocyte sodium and potassium pumps regulate Na+ and K+ levels and the chondrocyte resting potential [103]. Sodium and potassium gradients are established in chondrocytes and maintained by active ATP pumps [106,107,108]. Chondrocyte cell membrane α, β, and γ isoform sub-unit Na+ /K+ pump proteins have been identified [108,109,110]. Proteomic studies on the chondrocyte ‘‘surfaceome’’ have confirmed the presence of multiple Na+/K+ pump isoforms [102]. Perlecan stabilizes the pericellular environment of chondrocytes [10], promoting the establishment of Na+/K+ gradients that regulate chondrocyte behaviour. However, aggrecan, the major cartilage ECM proteoglycan, may limit the extent of these gradients. Na+/K+ are also counterions of aggrecan which regulate the osmotic and ionic environment of chondrocytes and equip cartilage with its hydration and weight-bearing properties [111]. Compression of cartilage results in an efflux of water from the cartilage and concentration of charge density due to aggrecan GAG side chains [112]. GAGs are electroconductive, thus compression of cartilage leads to the generation of an action potential that affects ion-streaming, resulting in membrane de-polarization and disruption of the Na+/K+ gradient formation at the chondrocyte cell surface [113]. Electrogenic Na+/K+ gradients at the chondrocyte cell surface may be short lived and re-formed during periods of cartilage un-loading. Piezoelectric properties of collagen in stressed weight-bearing and tensional connective tissues may also modulate Na+/K+ gradients [114,115].

### Perlecan Participates in Mechanotransductive Cartilage Regulation

Biomechanical measurement of individual chondrocytes and groups of chondrocytes demonstrate a progressive measurable decrease in stiffness in the pericellular matrix (PCM) of chondrocytes within a group. Relaxation of the enclosed chondrocyte PCM makes it more susceptible to influences from external stimuli and a greater susceptibility to proteolytic modification, which can further modulate cellular activity within cartilage and is evident during OA [21]. The PCM is a dynamic mechano-sensitive cell matrix interface that sends feedback cues back to the chondrocyte, which dynamically modulates its biosynthetic activity in response to perceived alterations in its biomechanical microenvironment, and a matrix is laid down in response to the altered chondrocyte environment to protect the chondrocyte from overloading [20]. Compared to the ECM, the PCM contains a relatively high proteoglycan concentration. The SLRP family members biglycan and decorin attach adjacent to the N-terminus of the type VI collagen triple helix, a major component of the chondron [116]. Perlecan interacts with type VI collagen in the PCM through multi-point attachments [7] and in hyaline and rudiment cartilage at all stages of joint development [9,25,26,117]. This PCM chondron capsule has mechanical properties which determine the stiffness of cartilage, and protects chondrocytes contained within it which respond to biomechanical ECM cues that regulate cartilage remodelling, repair, and development [18], and in OA [24].

## 5. Heparanase Influences Chondrogenesis and Osteogenesis

It is well known that cartilage has a poor ability to undergo self-repair. It has been suggested that the pericellular matrix of chondrocytes acts as an intrinsic basement membrane for this cell type [118]. Basement membranes are structures which finely tune cellular function and cell–matrix interactions. In chondrocyte monolayer cultures where HS levels are depleted by inclusion of heparanase in the culture medium, increased chondrocyte proliferative rates and GAG deposition occurs compared to control cultures [119]. 

Chondrocytes rapidly synthesise and activate pro-heparanase [87,120,121]. Heparanase in OA cartilage induces catabolic responses [121] inducing expression of the catabolic genes MMP-13 and a disintegrin and metalloproteinase with thrombospondin motifs (ADAMTS)-4 and secretion of active MMP-13, but also downregulates anabolic genes (ACAN, COL2A1) contributing to cartilage changes in OA [121]. A heparin mimetic, HP545, inhibits these effects [121]. Heparanase stimulates chondrogenesis and is upregulated in ectopic cartilage contributing to exostosis development [87]. Hereditary multiple exostosis patients carry heterozygous mutations in the HS-biosynthetic enzymes exostosin-1 and 2 (EXT1 or EXT2), partially explaining the depleted HS levels in these patients [122]. Heparanase is produced by most chondrocytes, however in normal cartilage growth plates from patients unaffected by hereditary multiple exostoses, it is produced only by the hypertrophic cells [87]. Treatment of murine mesenchymal chondroprogenitor limb bud cultures with heparanase-stimulated chondrogenesis and BMP signalling, cell proliferation, migration, and matrix deposition. A potent heparanase inhibitor, SST0001, strongly inhibits chondrogenesis [87]. Stimulation of ectopic cartilage formation by heparanase also upregulates BMP signalling and exostosis formation [88].

Sequential chondrogenis and osteogenesis occurs during endochondral bone formation, and requires the activity of heparin binding growth factors and their receptors. Heparanase expressed in the perichondrium, periosteum, and at the chondroosseous junction facilitates the transition of chondrogenic to osteogenic differentiation in the long bone growth plates during formation of endochondral bone [121]. Heparanase facilitates the laying down of bone at the chondroosseous junction through removal of perlecan–HS chains. The HS chains of perlecan prevent osteogenic cells from converting hypertrophic cartilage to bone [121]. Heparanase expression is elevated during osteogenic differentiation of rat marrow stromal cells and is also expressed by osteoblasts in culture, by osteoblasts at the chondro-osseous junction, and in fracture repair sites where it stimulates new bone formation. Perlecan expression is upregulated in hypertrophic growth plate chondrocytes; however, it is only when the HS is degraded or perlecan undergoes proteolytic degradation by MMP-13 at the chondro-osseous junction that bone formation occurs. Osteoblasts synthesise abundant levels of heparanase and the hypertrophic chondrocytes of the chondro-osseous junction contain abundant levels of MMP-13 that degrades perlecan in this region, releasing core protein peptides from perlecan domain-I and releasing HS. The elevated production of heparanase by osteoblasts and degradation of perlecan–HS releases bound growth factors such as the FGFs and VEGF165, which stimulate angiogenesis and promote bone formation [123,124,125].

### Heparanase Promotes Wound Repair and Tissue Remodelling

While the HS chains of perlecan have indispensable roles to play in embryogenesis and skeletogenesis, HS can also inhibit adult tissue repair processes. Degradation of HS in situ improves wound healing and tissue repair through the re-mobilisation of previously sequestered FGF-2 that was unavailable to promote tissue repair. Heparanase releases FGF-2 in tissues in an activated form which promotes wound healing [126,127]. Heparanase expression in osteoblastic cells also promotes bone formation and tissue remodelling at the osteochondral interface. Heparanase converts syndecan-1 shed from the cell surface to an activator of FGF-2. Similarly, intradermal injection of heparinase III removes HS from wound sites, accelerating the healing of diabetic ulcers that do not heal without this intervention. Selective removal of HS from cartilage perlecan or chemical inactivation of HS may also make this tissue more responsive to repair processes. Perlecan is essential to cartilage development [28]. HS chains in perlecan domain-1 interact with BMP-2, stimulating chondrogenic differentiation and bone formation [128].

The absence of HS chains in perlecan of the *Hspg2* exon 3 null mouse results in a phenotype seen in mice with mutations or deletions in EXT1 and EXT2 [15]. Upregulation of perlecan synthesis by hypertrophic growth plate chondrocytes promotes endochondral bone formation, but requires the degradation of perlecan–HS at the chondro-osseous junction before bone formation can occur. Osteoblasts synthesise abundant levels of heparanase and MMP-13 that degrade growth plate perlecan [129]. Cultured stem cells of a chondrogenic phenotype demonstrate a shift in differentiation to an osteogenic phenotype [130].

## 6. Internalisation of Cell Surface HS-PGs and Their Nuclear Translocation

Shuttling of extracellular HS-PGs into the nucleus occurs in a number of cell types [131,132,133]. Nuclear glypican in neurons and glioma cells [134], nuclear SDC-4 in cardiomyocytes [135], SDC-1 in fibrosarcoma cells, and SDC-2 in the nucleus of osteochondromas have all been documented. FGF-2 is transported to the nucleus of corneal stromal fibroblasts following injury by HS-PGs [134]. An upregulation in the expression of *Snail-1* occurs when SDC-1 is translocated to the nucleus of prostate cancer cells and may result in inhibition of histone acetyltransferase activity and chromatin compaction, resulting in decreased transcription factor access to DNA and resultant alterations in gene expression, cell cycle control, and cellular proliferation normally provided by the transcriptional machinery. GAGs are potent inhibitors of histone acetyltransferases [136]. Acetylation and deacetylation are well known modulators of nucleosomal histones and chromatin structure, that affect transcription activity and gene expression. Opposing effects of histone acetyltransferases and deacetylases on histones affects chromatin structure, thus they need to be tightly controlled. Acetylation is a common modification of histones, and a key way to modulate chromatin structure and gene transcription.

**Figure 5:**
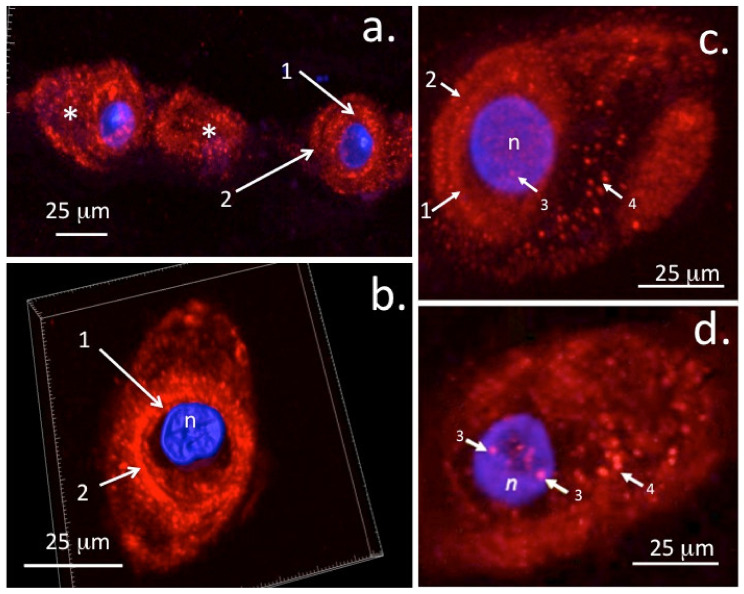
Immunolocalisation of perlecan in ovine AF and NP chondrons using laser scanning confocal microscopy and 3D rendered image stacks. Nuclear DAPI counterstaining is shown in blue. Fluorescent perlecan immunolocalisations were undertaken as described in [140]. A string of outer AF cells (**a**) and 3D reconstructions of perlecan in an NP chondron with 3D volume indicated by white boundary box (**b**). Immunolocalisation of perlecan in a stacked confocal image of an NP chondron (**c**) and in a 0.5 µm single z-stack image depicting punctate nuclear perlecan deposits (**d**). These deposits are obscured in the stacked image (**c**) by overlying tissue. Key: 1. Pericellular matrix, 2. Type VI Collagenous capsule, 3. Nuclear deposits of perlecan, 4. Vesicular perlecan transported out of the cell into the chondron matrix (*). n = nucleus. Figure reproduced under Open Access CC-BY-SA licence from [137] © the authors 2020.

The presence of perlecan as a nuclear component in IVD cells [137] opens up further possible regulatory roles in chondrocytes (Figure 5). It is not known if differences exist in the translocation of perlecan to the nucleus of chondrocytes in different cartilage types that would afford differential regulatory properties in some tissues. As seen in this review, rudiment and growth plate cartilages undergo cellular proliferation, matrix expansion, and chondrocyte maturation to hypertrophy, leading to the establishment of the primary and secondary ossification centres [17], endochondral ossification, bone formation, and extension of the axial and appendicular skeleton [138]. Chondrocytes in articular cartilage, however, do not undergo such changes and have a notoriously poor ability to participate in cartilage repair processes, thus their activity appears to be firmly held in a homeostatic state. The potential role of nuclear perlecans in the inhibition of histone acetyl transferase resulting in chromatin condensation and inhibition of transcriptional activity may be a potential reason why mature articular chondrocytes do not proliferate, make significant ECM components, or participate in repair processes. Studies have yet to be undertaken to provide answers to these questions. A recent study, however, has shown that articular chondrocytes do not totally lose their capacity to undergo cartilage repair. In some contexts, articular chondrocytes can revert to an embryonic phenotype and can recapitulate ECM changes seen in embryonic cartilage development where perlecan is a prominent ECM component [139]. It is important that the properties of nuclear perlecan be ascertained, as this may reveal a novel therapeutic area which can be manipulated to improve cartilage repair.

### 6.1. Nuclear HS-proteoglycans and Their Regulation by Heparanase

Many cell types have been shown to contain heparin, HS, or specific HS-PGs in their nuclei; these include astrocytes, endothelial cells, keratinocytes, hepatocytes, fibroblasts, chondrocytes, intervertebral disc cells, and neurons [132,137]. Nuclear HS has been proposed to regulate the cell cycle, cell proliferation, and transcription [132]. GAGs are potent inhibitors of histone acetyltransferases, with HS being the most potent inhibitor [136], thus perinuclear HS-PGs may potentially regulate acetylation/deacetylation of histones, modifying chromatin structure and affecting the access of transcription factors such as *Snail-1* to DNA. Nuclear HS-PGs can potentially regulate DNA topoisomerase I activity and its essential roles untangling DNA supercoils in transcription, DNA replication and DNA re-annealment of DNA after strand breakage in re-combination, and chromosomal condensation and disentanglement of intertwined DNA strands during mitosis [141,142]. Heparanase associated with chromatin [143] can further modify HS-PG structure and levels in the nucleus [144] regulating gene expression.

### 6.2. Chromatin Re-Organisation Provided by Histone Acetylation/Deacetylation Alters Nucleosome Structure That Can Potentially Impact on the Regulation of Chondrocyte Gene Expression

Regulation of histone acetyl transferases (HATs) and histone deacetylases (HDACs) affects physiological and pathological cellular processes [145,146], regulating gene expression by opening or closing the chromatin structure to allow transcriptional access to regulate cell cycle progression, cellular proliferation, and differentiation. The ability of HS and HS-PGs to regulate the enzymes that control chromatin organization thus has implications in the control of gene regulation [132]. Similar roles for nuclear perlecan can thus be envoked [137]. HDAC4 represses chondrocyte hypertrophy and prevents endochondral bone development through inhibition of myocyte-specific enhancer factor 2C (MEF2C) and runt-related transcription factor 2 (RUNX2) [147]. HDAC4 promotes chromatin condensation, denying transcriptional factor access to DNA, repressively regulating chondrocyte maturation and the attainment of a pre-hypertrophic state. Deletion of HDAC4 results in premature calcification of cartilage [148]. This is supported by HDAC4 null mice which have severely runted frames, shortened growth plate hypertrophic zones, enhanced vascular invasion, and cartilage mineralization. *MMP-13, Runx2, OPG, CD34,* and *Wnt5a* are all downregulated and type X collagen elevated in HDCA4 null mice. Adenoviral-mediated transduction of HDAC4 ameliorates disease progression [149] and lowers MEF2C and RUNX2 activity and cartilage degeneration [150,151]. HDAC4 and Snail-1 thus both potentially aid in permanent cartilage homeostasis. An elevation in HDAC2 activity in OA patients enhances cartilage degradation and represses cartilage-specific gene expression [152]. MicroRNAs have been developed to inhibit HDAC2 and HDAC3 [153,154,155,156,157] and regulate aggrecanase-1 and aggrecanase-2 activity in IL-1β-induced catabolic changes in human articular chondrocytes [155]. Future studies on nuclear perlecan are warranted to fully determine its potential impact on gene regulation [156]. Available evidence indicates that this may represent a new therapeutic window in chondrocyte regulation.

Interaction of nuclear/perinuclear perlecan with nuclear FGF-1, -2, and FGFR-1 may occur in a similar way to in the extracellular environment [157], however this cannot be assumed and studies need to be undertaken to assess this possibility. Quiescent cells do not contain nuclear FGFR-1, but cells treated with FGF-2 display a dose- and time-dependant increase in nuclear FGFR-1. Biotin-labelled cell surface FGFR-1 localises in a nuclear fraction of FGF-2-treated cells, thus FGFR-1 is translocated to the nucleus from the cell surface [138]. Four intracellular non-signalling nuclear FGFs interact with voltage-gated ion channels to regulate intracellular sodium levels. A single FGF-2 transcript can be translated into an 18kDa low molecular weight isoform that is secreted, and four 32-34 kDa nuclear isoforms [158,159,160,161] interact with ion channels and stem cell renewal [162]. Nuclear FGFR and polypeptide growth factor signalling regulate skeletal development [163,164]. FGFRs interact with chromatin remodelling proteins to alter the epigenetic state and transcriptional status of target genes [164].

### 6.3. Potential Regulatory Roles of Nuclear Perlecan on Mechanosensory Transcription Factor Activity

The proposal that nuclear perlecan could potentially influence chromatin and nucleosomal structure, thereby influencing the activity of transcription factors that regulate chondrocyte metabolism, is supported by studies on agrin which have shown that this HS-PG is linked to mechanotransduction regulating the nuclear mechanosensors YAP and TAZ [163,164,165,166,167,168]. The Hippo pathway effector molecules YAP (yes-associated protein 1) and TAZ (WWTR1), a 14-3-3 binding protein with a PDZ binding motif, are nuclear mechano-sensors [165]. Interaction between Agrin and YAP co-activator transduces matrix and cellular signals with Lrp4/MuSK receptor-mediated signalling pathways [166]. TAZ is a cell sensor that signals cues generated by high cell density and ECM stiffness. TAZ overexpression in breast and papillary thyroid carcinoma may promote tumour development [168]. Perlecan is also upregulated in some tumours [169] and has roles in mechanosensory cell–matrix communication, extracellular matrix stabilisation, and mechanoregulation. Nuclear perlecan may also have novel roles similar to YAP/TAZ that have yet to be determined [20]. YAP/TAZ participate in integrin-mediated cell–ECM cell signalling and mechano-transduction [167]. Pathologic changes in tissues that occur in cancer and cardiovascular disease perturb cellular behaviour, leading to stiffening of the glycocalyx and ECM due to tissue fibrosis. Perlecan also has roles to play in the promotion of tissue fibrosis [170]. Increasing evidence suggests that a dense PG-rich glycocalyx promotes malignant transformation, and the aggressiveness of cancer types and the response of tumour cells to therapeutic treatment regimens [171]. Perlecan is upregulated in certain tumour types. Parallels are thus evident between perlecan and agrin not only in the regulation of mechanosensory processes but also in pathologic changes seen in some tissues.

## 7. Concluding Remarks

Perlecan promotes cellular proliferation and differentiation and matrix deposition, which drives tissue expansion and skeletal development in the transitional rudiment and growth plate cartilages, whereas in the permanent cartilages tissue dimensions are maintained through control of proliferation, differentiation, and matrix deposition to provide tissue homeostasis but not tissue expansion. Perlecan promotes tissue development through growth factors and morphogens, while in the permanent cartilage growth factor bio-availability is controlled to maintain tissue homeostasis. In tissues where extracellular gradients of perlecan, Wnt, and Hh occur, such as in the rudiment and growth plate cartilages, cellular proliferation, differentiation to hypertrophy, matrix deposition, tissue expansion, and tissue morphogenesis occur, while in the permanent cartilages these gradients do not occur and articular cartilage is held in homeostatic balance. Thus, perlecan displays differing cell-regulatory properties depending on its distribution in tissues. Perinuclear and nuclear perlecan may also regulate chondrocytes. HS is a potent inhibitor of histone acetyltransferases resulting in DNA compaction. Perinuclear perlecan may also modulate *Snail-1* activity and promote FGF-FGFR3 signalling in pre-hypertrophic growth plate chondrocytes. *Snail-1* represses the transcription of *Acan* and *Col2A1* in chondrocytes through human histone deacetylase 1 and 2. This is a novel area of cellular regulation requiring further exploration in cartilage regeneration or repair biology. The recent demonstration of perlecan as a nuclear component suggests perlecan can also exert regulatory control directly at the gene level. Further studies are thus warranted to further explore this aspect of perlecan’s biology. Perlecan’s roles as a ubiquitous, multifunctional, and pleomorphic molecule is of considerable biological importance. A greater understanding of perlecan’s diverse biological roles and functional repertoires during cartilage development and maturation as a precursor to expansion of the axial and appendicular skeleton will yield invaluable insights as to how this impressive proteoglycan could potentially be utilized in cartilage repair biology. Cartilage is a tissue with a notoriously poor intrinsic repair potential. Recent studies, however, have nevertheless emphasized the relevance of perlecan in cartilage repair strategies [139,172], and now that recombinant perlecan is available in the laboratory [173], exciting possibilities exist for the exploration of repair strategies for this most intransigent of tissues which were not previously possible.

## Figures and Tables

**Table 1:** Perlecan interactive ligands with specific domains.

Domain I	Domain II	Domain III	Domain IV	Domain V
Laminin-I	VLDL	FGF-7	Nidogen-1	Nidogen-1
Collagen IV	LDL	FGF-18	Nidogen-2	Fibulin-2
Collagen V	Fibrillin-1	FGF-BP	Fibronectin	β1-integrin
Collagen VI	Wnt	PDGF	Collagen IV	α-dystroglycan
Collagen XI	Hedgehog	WARP	PDGF	FGF-7
Fibronectin		Collagen VI	Fibulin-2	Endostatin
PRELP		Tropoelastin	Collagen VI	ECM-1
WARP			Tropoelastin	Collagen VI
Fibrillin-1				Progranulin
Thromobospondin				Acetylcholinesterase
FGF-1, 2, 7, 9, 18				α2 β1 integrin
BMP-2				Tropoelastin
PDGF				
VEGF				
IL-2				
Hedgehog				
Ang-3				
Heparanase				
Activin-A				
G6b-B-R				
Histone-H1				

Abbreviations: PRELP, proline/arginine-rich end leucine-rich repeat protein; WARP, von Willebrand factor A domain-related protein; FGF, fibroblast growth factor; FGF-BP, FGF binding protein; BMP, bone morphogenetic protein; PDGF, platelet derived growth factor; VEGF, vascular endothelial cell growth factor; IL, interleukin; Ang, angiotensin; G6b-B-R, Megakaryocyte and platelet inhibitory receptor G6b;VLDL, very low density lipoprotein; LDL, low density lipoprotein; Wnt, Wingless/Int; ECM-1, Extracellular matrix protein-1.

## Data Availability

All data is presented within the cited studies.

## Abbreviations

ADAMTS, A Disintegrin and Metalloproteinase with Thrombospondin motifs; AF, Annulus fibrosus; BMMSCs, Bone marrow mesenchymal stromal cells; BMP, Bone morphogenetic protein; CS, chondroitin sulphate; ECM, extracellular matrix; EGF, epidermal growth factor; ERK, Extracellular signal-related kinase; ESCs, embryonic stem cells; EXT, exostosin; FGF, Fibroblast growth factor; FGFR, Fibroblast growth factor receptor; GlcA, Glucuronic acid; GlcNAc, N-Acetyl glucosamine; GAG, glycosaminoglycan; Hh, Hesgehog; HME, hereditary multiple exostosis; HS, heparan sulphate; HSPGs, heparan sulphate proteoglycans; HUAEC, human umbilical artery endothelial cell; IdoA, iduronic acid; IVD, intervertebral disc; KS, keratan sulphate; MAPK, Mitogen activated protein kinase; MMP, matrix metalloprotease; MSC, mesenchymal stem cell; NP, nucleus pulposus; OA, osteoarthritis; PCM, pericellular matrix; PDGF, platelet derived growth factor; PI3, phosphatidylinositol-3-kinase; PDK, Phosphoinosotide-dependent kinase; Ras, a contraction of the term ‘Rat sarcoma’, the tumour where the first gene of this family of oncogenes was identified; RTK, receptor tyrosine kinase; SEVI, Semen-derived enhancer of viral infection; SFRPI, secreted frizzled-related protein-1; VGP, vertebral growth plate; Widr, A human colon carcinoma cell line; Wnt; derived term from a mouse proto-oncogene (Int1, integration-1 protein) and the Wingless protein originally identified in Drosophila. Wnt is a condensation of the int and Wg protein terms and stands for Wingless-related integration site. YAP, yes-associated protein 1. TAZ (WWTR1), a 14-3-3 binding protein with a PDZ binding motif.
